# Abdominal surgery prior to chronic psychosocial stress promotes spleen cell (re)activity and glucocorticoid resistance

**DOI:** 10.1038/s41598-020-63419-4

**Published:** 2020-04-24

**Authors:** Sandra Foertsch, Dominik Langgartner, Stefan O. Reber

**Affiliations:** Laboratory for Molecular Psychosomatics, Department of Psychosomatic Medicine and Psychotherapy, Ulm University, Ulm, Germany

**Keywords:** Chronic inflammation, Risk factors

## Abstract

There is convincing evidence from different mouse models that chronic psychosocial stress promotes splenomegaly, basal and lipopolysaccharide (LPS)-induced *in vitro* splenocyte activation and insensitivity towards glucocorticoids (GC) in *in vitro* LPS-treated splenocytes. However, we just recently showed, employing the chronic subordinate colony housing (CSC) paradigm, that bite wounds received during stressor exposure drive these stress-induced spleen changes. As skin wounds induced by planned surgery or physical trauma are more adequately reflecting what chronically stressed humans are likely to experience, it was the objective of the present study to investigate whether abdominal surgery prior to stressor exposure also promotes respective stress-induced spleen effects in the absence of any bite wounds. In line with our hypothesis, abdominal surgery prior to CSC induced splenomegaly, increased *in vitro* cell viability under basal and LPS conditions as well as the delta response to LPS (LPS – basal), and promoted the inability of isolated splenocytes to respond with a decreased cell viability to increasing concentrations of corticosterone following LPS-stimulation *in vitro*. Together with previous data, these findings demonstrate that physical injury, either in form of received bite wounds during stressor exposure or in form of abdominal surgery prior to stressor exposure, promotes the development of splenic immune activation and GC resistance.

## Introduction

In our previous study, we identified bite wounds to be a critical factor in the development of splenomegaly and splenic glucocorticoid (GC) insensitivity, as well as to facilitate spleen cell activation following exposure to the chronic subordinate colony housing (CSC) paradigm^1^. The latter is a pre-clinically validated mouse model for social stress-induced posttraumatic stress disorder (PTSD)^2–4^. Moreover, it reliably promotes local and systemic immune activation, accompanied by increased general and social anxiety and a variety of somatic comorbidities^2,5–7^. The development of bite wound-dependent splenomegaly, despite a comparable number of splenocytes between single-housed control (SHC) and CSC mice, was likely due to an altered composition of splenocytes. This was indicated by an increase in larger CD11b^+^ cells and a decrease in smaller CD3^+^/CD4^+^ T- and CD19^+^ B-cells^8^. The dependence of *in vitro* splenic GC insensitivity on the occurrence of bite wounds was indicated by the inability of *in vitro* lipopolysaccharide (LPS)-stimulated CD11b^+^ splenocytes, but not splenocytes depleted of CD11b^+^ cells, to respond to the immunosuppressive actions of corticosterone (CORT), the main effector molecule of the hypothalamus-pituitary-adrenal (HPA) axis in rodents^1,2^. Increased spleen cell activation following CSC exposure was indexed by an increased *in vitro* cell viability of isolated splenocytes from CSC vs. SHC mice, both in the presence and absence of LPS^1^. The latter effects were more pronounced in isolated CD11b^+^ splenocytes, but also detectable in CD11b^−^ spleen cells. Therefore we concluded, that the enhanced spleen cell (re)activity following CSC is not critically dependent on, but facilitated by, bite wound-induced immigration of activated and GC resistant CD11b^+^ cells into the spleen^1^. Notably, detailed behavioral analyses during CSC exposure revealed a positive correlation between re-active coping behavior and the severity of bite wounds. In turn, the assessed bite score highly correlated with the dimension of splenic GC resistance, as well as with basal and LPS-induced *in vitro* splenocyte viability^1^. Importantly, social stress-induced splenomegaly, spleen cell activation and splenic GC insensitivity are not CSC paradigm-specific phenomena, as they have been also reported following other social stress paradigms, like for example in rodents exposed to the social disruption paradigm (SDR)^9–13^.

Thus, considering the currently available literature, previous studies of our group and from others propose that any type of psychological stressor is able to promote proliferation and emigration of CD11b^+^ myeloid cells via sympathetic signaling locally in the bone marrow^5,14–16^. If the stressor is of social nature allowing direct physical interaction between the individuals, these circulating CD11b^+^ cells in a bite wound-dependent manner accumulate in the spleen of socially defeated rodents^1,16,17^. The latter is accompanied by increased susceptibility to endotoxic shock^18^, which is at least in part due to increased splenic expression of toll-like receptors (TLR)2 and 4^19^, basal and/or LPS-induced *in vitro* cell viability^1,10,12^ and production of interleukin (IL)-6, IL-1β and tumor necrosis factor (TNF)-α^18,20,21^, as well as development of GC resistance^1,2,10,12,13,16^.

As skin wounds induced by planned surgery or physical trauma are more adequately reflecting what chronically-stressed humans are likely to experience, it was the objective of the present study to investigate whether abdominal surgery prior to stressor exposure also promotes stress-induced splenomegaly, spleen cell activation and development of splenic GC resistance in the absence of any bite wounds.

## Methods and Materials

### Animals

All mice used in this study were purchased from Charles River (Sulzfeld, Germany). Male C57BL/6 N mice, weighing 17–21 g, were used as experimental mice and male CD-1 mice, weighing 30–35 g, were used as dominant resident mice. Standard laboratory conditions (12 h light/12 h dark cycle, lights on at 07:00 am, 22 °C, 60% humidity) were applied to all mice as well as free access to tap water and standard mouse diet. All animal experiments performed for this study were in compliance with international regulations for the care and use of laboratory animals (ARRIVE guidelines and EU Directive 2010/63/EU for animal experiments) with the approval of the Local Ethical Committee (No. 1216, Regierungspräsidium Tübingen, Germany).

### Experimental procedure

**Figure 1:**
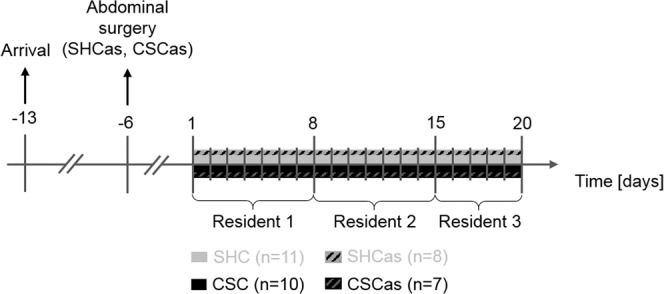
Schematic representation of the experimental timeline. Following arrival at day −13, all mice were housed individually for at least one week. Abdominal surgery was performed at day −6, one week prior to the start of the chronic subordinate colony housing (CSC) paradigm in one set of CSC and single-housed control (SHC) mice (SHCas; CSCas). On day 1 of the CSC paradigm, experimental CSC mice were housed together with a dominant male CD-1 mouse for 19 consecutive days to induce chronic psychosocial traumatization. To avoid habituation, CSC mice were introduced into the home cage of a novel aggressor mouse on days 8 and 15. Except for a weekly change of bedding, SHC mice remained undisturbed in their home cages for 19 consecutive days. All experimental mice were euthanized in the morning of day 20.

All male C57BL/6 N mice were either single-housed for control or chronically stressed by 19-day exposure to the CSC paradigm (Fig. 1). One set of SHC (n = 11) and CSC (n = 10) mice was kept singly for two weeks after delivery (day -13) from the supplier until the onset of the SHC and CSC procedure (day 1). The here presented spleen data of this set of mice was also considered in the data pool published recently^1^, where we pooled various different CSC experiments conducted during the last 5 years to obtain a large n number for correlational analyses. Another set of SHC (SHCas; n = 8) and CSC (CSCas; n = 7) mice was kept singly for two weeks after delivery (day -13) from the supplier before the start of the SHC and CSC procedure (day 1), but underwent abdominal surgery for G2 HR E-mitter (Starr Life Sciences Corp., Oakmont, PA; USA) implantation on day -6. The physiological parameters (i.e. body -, relative adrenal-, and relative thymus weight) of this set of mice, but not the data presented in the current manuscript, have already been published recently^22^. Euthanization of all experimental mice occurred in the morning of day 20 followed by the assessment of spleen parameters. Due to the limited number of E-mitters available, the indicated group numbers of all experimental groups were generated in three subsequent experiments.

### Chronic subordinate colony housing (CSC) paradigm

The CSC paradigm was performed as previously described^2,3,16,22–24^ with minor modifications. Briefly, all mice were housed individually for two  weeks after delivery from the supplier. Abdominal surgery was performed one week prior to the start of the CSC paradigm. In the morning of day 1 of the CSC paradigm, experimental CSC mice were introduced into the home cage of a dominant male CD-1 resident and kept under chronic subordination for 19 consecutive days. Habituation to the dominant aggressor mouse was avoided by introducing the four CSC mice into the home cage of a novel, unfamiliar CD-1 resident in the morning of days 8 and 15. SHC mice were kept undisturbed in their home cages for 19 consecutive days, except for the change of bedding once a week. Our own previous data indicate that single housing, but not group housing, represents the most appropriate control group for the CSC paradigm^25^.

### Abdominal surgery: E-mitter implantation

The G2 HR E-mitters (Starr Life Sciences Corp., Oakmont, PA; USA) were implanted into the abdominal cavity of SHCas and CSCas mice as previously described^22^.

### Assessment of dermal and subdermal bite wound severity

The severity of bite wounds on the body as well as on the skin was assessed as previously described^1^. Briefly, the skin (with fur attached) of CSC and CSCas mice was removed from the body following decapitation. Pictures depicting both, the derma and subderma of each mouse were taken. A standardized grid with 4 ×5 squares (each square 180 ×240 pixels) was digitally placed on the skin and the body, respectively. The quality and quantity of received bite wounds was scored by an experienced  observer blind to treatment according to a recently developed score, considering both the area and intensity of wounds on the skin (dermal) and the body (subdermal)^1^. The total bite score represents the added score of the dermal and subdermal score of each mouse and possible scores range from 0 to 300.

### Determination of spleen weight

Following CO_2_ anesthesia and immediate decapitation in the morning of day 20, spleens were removed and pruned of surrounding fat, followed by weighing and storage in ice-cold Hanks´ balanced salt solution (HBSS) until splenocyte isolation.

### *In vitro* splenocyte GC sensitivity assay

Following euthanization of all mice of the respective experiment, splenocytes were isolated from the spleen of each mouse as previously described^1,2^. Briefly, following homogenization of the spleens, erythrocytes were removed from the single-cell suspension by 2-min incubation with lysis buffer (155 mM NH_4_Cl, 10 mM KHCO_3_, 10 mM EDTA). The lysis was stopped by addition of HBSS containing 10% of heat-inactivated fetal calf serum (FCS). Afterwards, the cell suspension was again passed through a 70 µm nylon cell strainer and washed, followed by assessment of the number of isolated splenocytes by use of a cell counter (TC-20, Bio-Rad Laboratories, Munich, Germany). Then, adjustment of the cell concentrations to 5 × 10^6^ cells/ml was performed in RPMI containing 10% FCS, 50 U/ml penicillin and 50 µg/ml streptomycin. Stimulation of the isolated splenocytes occurred with LPS (Escherichia coli O111:B4; final concentration: 1 µg/ml; Sigma-Aldrich, Deisenhofen, Germany) or splenocytes remained untreated for the assessment of basal *in vitro* activity. GC sensitivity of isolated splenocytes derived from SHC and CSC mice was determined by treatment of unstimulated as well as LPS-stimulated cells with different CORT (Sigma-Aldrich, Deisenhofen, Germany) concentrations (final concentrations: 0, 0.005, 0.05, 0.1, 0.5 and 5 µM respectively) diluted in 95% ethanol. Stimulation of 2.5 × 10^5^ cells/well was performed in flat-bottom 96-well plates (final volume 100 µl/well) and were incubated for 48 h (37 °C, 5% CO_2_). Afterwards, cell viability was measured by use of a commercially available colorimetric assay (CellTiter 96 Aqueous One Solution Assay, Promega, Madison, WI) and the absorbance (optical density (OD)) of each well was measured at 450 nm using an enzyme-linked immunosorbent assay (ELISA) plate reader (Fluostar Optima, BMG Labtech, Offenburg, Germany). The delta cell viability was calculated by subtracting the OD of unstimulated cells for a given CORT concentration from the corresponding LPS-stimulated well.

### Statistics

Statistical comparison was performed by use of the software package IBM SPSS statistics (version 25.0). Normal distribution of all groups per data set was calculated by employing Kolmogorov-Smirnov test using Lilliefors‘ correction. In the case that more than 75% of groups per data set were identified to be normally distributed, Grubbs test was performed to identify outliers, which were then excluded from further statistical analyses. Normally distributed data sets were further analysed using parametric statistics, i.e. parametric Student’s *t*-test (one factor, two independent samples), one-way analysis of variance (ANOVA; one factor, two or more independent samples), two-way ANOVA (two factors, two or more independent samples), linear mixed models (LMM; two factors, two or more dependent samples). For tests considering more than two samples, a significant main effect was followed by *post-hoc* analysis using Bonferroni pairwise comparison. Non-parametric statistics was applied for non-normally distributed data sets, i.e. Mann-Whitney-U-test (one factor, two independent samples), Kruskal Wallis test (one factor, two or more independent samples), Friedman ANOVA (one factor, more than two dependent samples). Normally distributed data are presented as bars (mean + SEM) with corresponding dot plots. Non-normally distributed data are presented as box plots. Solid line represents the median, dashed line represents the mean for each data set. Lower box indicates 25^th^_,_ upper box indicates 75^th^ percentile. If n > 8 per group, 10^th^(lower error bar), and 90^th^ percentile (upper error bar) as well as possible outliers beyond the percentiles (indicated by closed circles) are shown. The level of significance was set at *P* ≤ 0.05.

## Results

### Abdominal surgery prior to stressor exposure is critical for CSC-induced splenomegaly

**Figure 2:**
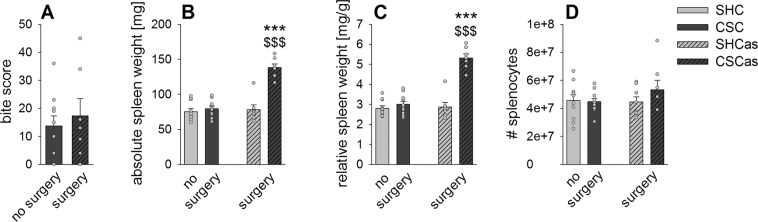
Abdominal surgery prior to CSC is critically required for development of CSC-induced splenomegaly. (**A**) Bite score of mice exposed to 19 days of chronic subordinate colony housing (CSC) either without (no surgery; CSC; n = 10) or with abdominal surgery (surgery; CSCas; n = 7) one week prior to the start of the stress protocol. (**B**,**C**), Absolute (**B**) and relative (**C**) spleen weight of CSC and CSCas mice as well of their respective single-housed control (SHC) mice (SHC: n = 11; SHCas: n = 8). (**D**), Number of splenocytes per mouse in all experimental groups. Data sets are presented as bar graphs (mean + SEM) with corresponding dot plots. ***P < 0.001 vs. respective SHC. ^$$$^P ≤ 0.001 vs. respective no surgery group.

Although there was no statistically significant difference in the bite score between CSC and CSCas mice (Fig. 2A), absolute (Fig. 2B, Table 1) and relative (Fig. 2C, Table 1) spleen weight was significantly increased in CSCas mice compared with both SHCas and CSC mice. Total number of splenocytes per animal was not affected (Fig. 2D).

**Table 1:** Relevant Statistical Effects.

*Statistical Effects for Figure 2*			
B) Absolute spleen weight	Factor stress x surgery	F_1, 31_ = 33.158, P ≤ 0.001	
	Post hoc	CSCas vs. SHCas	P ≤ 0.001
		CSCas vs. CSC	P ≤ 0.001
C) Relative spleen weight	Factor stress x surgery	F_1, 31_ = 42.853, P ≤ 0.001	
	Post hoc	CSCas vs. SHCas	P ≤ 0.001
		CSCas vs. CSC	P ≤ 0.001
***Statistical Effects for Figure 3***			
A) Cell viability	Factor stress	F_1, 30.212_ = 6.734, P = 0.014	
	Post hoc	basal: CSCas vs. SHCas	P = 0.006
		LPS: CSCas vs. SHCas	P ≤ 0.001
	Factor surgery	F_1, 30.212_ = 8.796, P = 0.006	
	Post hoc	basal: CSCas vs. CSC	P = 0.003
		LPS: CSCas vs. CSC	P ≤ 0.001
	Factor stimulus	F_1, 29.398_ = 586.290, P ≤ 0.001	
	Post hoc	All groups basal vs. LPS	P ≤ 0.001
B) Delta cell viability	Factor stress	F_1, 32_ = 4.921, P = 0.034	
	Post hoc	CSCas vs. SHCas	P = 0.014
	Factor surgery	F_1, 32_ = 7.812, P = 0.009	
	Post hoc	CSCas vs. CSC	P = 0.004
***Statistical Effects for Figure 4***			
A) Relative basal cell viability	Factor CORT	SHC: Χ^2^(5, 11) = 48.610, P ≤ 0.001	
		CSC: Χ^2^(5, 10) = 44.514, P ≤ 0.001	
	Post hoc	Both: 0.5 vs. 0 CORT	P ≤ 0.001
		Both: 5 vs. 0 CORT	P ≤ 0.001
B) Relative basal cell viability	Factor CORT	SHCas: Χ^2^(5, 8) = 31.357, P ≤ 0.001	
		CSCas: Χ^2^(5, 7) = 33.204, P ≤ 0.001	
	Post hoc	SHCas: 0.5 vs. 0 CORT	P = 0.020
		SHCas: 5 vs. 0 CORT	P = 0.008
		CSCas: 0.5 vs. 0 CORT	P = 0.003
		CSCas: 5 vs. 0 CORT	P ≤ 0.001
Separate MWU	0.1 µM CORT	CSCas vs. SHCas	P = 0.004
C) relative delta cell viability	Factor CORT	SHC: Χ^2^(5, 11) = 49.078, P ≤ 0.001	
		CSC: Χ^2^(5, 10) = 43.486, P ≤ 0.001	
	Post hoc	SHC: 0.1 vs. 0 CORT	P = 0.002
		CSC: 0.1 vs. 0 CORT	P = 0.008
		Both: 0.5 vs. 0 CORT	P ≤ 0.001
		Both: 5 vs. 0 CORT	P ≤ 0.001
Separate MWU	0.1 µM CORT	CSC vs. SHC	P = 0.036
D) relative delta cell viability	Factor CORT	SHCas: Χ^2^(5, 8) = 31.429, P ≤ 0.001	
		CSCas: Χ^2^(5, 7) = 12.714, P=0.026	
	Post hoc	SHCas: 0.1 vs. 0 CORT	P ≤ 0.001
		SHCas: 0.5 vs. 0 CORT	P = 0.002
		SHCas: 5 vs. 0 CORT	P = 0.003
Separate MWU	0.05 µM CORT	CSCas vs. SHCas	P = 0.002
	0.1 µM CORT	CSCas vs. SHCas	P ≤ 0.001
	0.5 µM CORT	CSCas vs. SHCas	P ≤ 0.001
	5 µM CORT	CSCas vs. SHCas	P = 0.002

### GC insensitivity following psychosocial stress depends on surgery prior to stressor exposure

**Figure 3:**
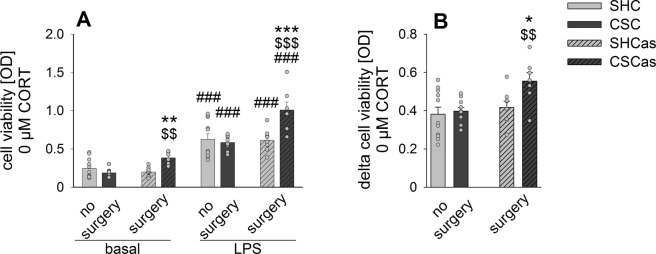
Abdominal surgery prior to CSC exposure is critically required for CSC-induced enhancement of splenic immune (re)activity. (**A**) Cell viability of isolated spleen cells under unstimulated (basal) or lipopolysaccharide (LPS)-stimulated conditions from mice exposed to 19 days of chronic subordinate colony housing (CSC) and single-housed controls (SHC) that did (SHCas, n = 8; CSCas, n = 7) or did not (SHC, n = 11; CSC, n = 10) undergo abdominal surgery one week prior to start of the stress protocol. (**B**), Delta cell viability (LPS minus basal) of isolated spleen cells from all experimental groups. Data sets are presented as bar graphs (mean + SEM) with corresponding dot plots. *P < 0.05, **P < 0.01, ***P < 0.001 vs. respective SHC. ^###^P ≤ 0.001 vs. respective basal condition. $$ P < 0.01, ^$$$^P ≤ 0.001 vs. respective no surgery group.

Statistical analysis of the cell viability of isolated splenocytes cultured *in vitro* under basal or LPS-stimulated conditions for 48 h (Fig. 3A, Table 1) revealed that basal spleen cell viability in CSCas mice was significantly higher compared to both SHCas and CSC mice. As expected, LPS stimulation increased spleen cell viability compared to the respective basal conditions in all groups assessed. Moreover, LPS-induced spleen cell viability in CSCas mice was significantly higher than in both SHCas mice and CSC mice. Delta cell viability (LPS minus basal; Fig. 3B, Table 1) was also  significantly increased in CSCas compared to both SHCas and CSC splenocytes.

**Figure 4:**
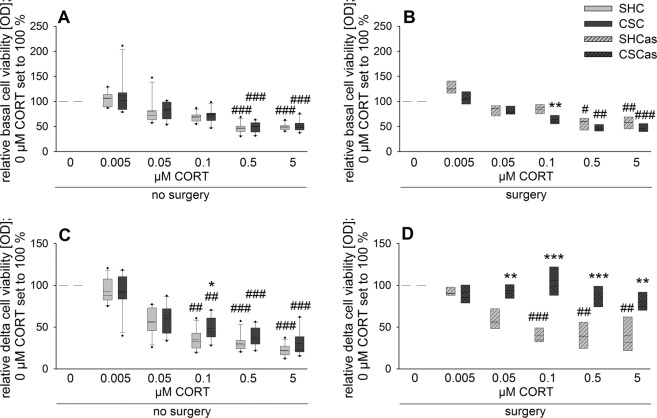
CSC-induced splenic GC insensitivity critically depends on abdominal surgery prior to CSC and presence of LPS during *in vitro* stimulation. (**A**,**B**), Relative basal cell viability of isolated spleen cells (0 µM corticosterone (CORT) set to 100%) in response to increasing (0–5 µM) concentrations of CORT. Splenocytes were isolated from mice exposed to 19 days of chronic subordinate colony housing (CSC) or single housing (SHC) that either did (SHCas, n = 8; CSCas, n = 7; (**B**)) or did not (SHC, n = 11; CSC, n = 10; (**A**)) undergo abdominal surgery one week prior to the start of the stress protocol. (**C**,**D**), Relative delta (LPS minus basal) cell viability of isolated spleen cells (0 µM CORT set to 100%) from SHC and CSC (**C**) as well as SHCas and CSCas (**D**) mice in response to increasing (0–5 µM) CORT concentrations. Data sets are presented as box plots. Solid line represents the median, dashed line represents the mean for each data set. Lower box indicates 25^th^, upper box indicates 75^th^ percentile. If n > 8 per group, 10^th^ (lower error bar), and 90^th^ percentile (upper error bar) are shown. *P < 0.05, **P < 0.01, ***P < 0.001 vs. respective SHC. ^#^P < 0.05, ^##^P < 0.01, ^###^P < 0.001 vs. respective 0 µM CORT condition.

In the absence of LPS, relative basal cell viability was sensitive to the immunosuppressive effects of CORT, independent of abdominal surgery and CSC exposure (Fig. 4A,B). This was indicated by a significantly decreased relative cell viability at 0.5 and 5 µM CORT compared to respective 0 µM CORT in SHC and CSC mice (Fig. 4A, Table 1) as well as in SHCas and CSCas (Fig. 4B, Table 1). Relative cell viability of CSCas vs. SHCas splenocytes was further decreased at 0.1 µM CORT.

In line with respective basal findings, relative delta cell viability (LPS minus basal; 0 µM CORT set to 100%) in splenocytes from mice that did not undergo abdominal surgery prior to the CSC or SHC (Fig. 4C, Table 1) also was significantly decreased in SHC and CSC mice at 0.1, 0.5 and 5 µM CORT when compared to the respective 0 µM CORT condition. Separate MWU testing revealed a significantly higher relative delta cell viability in splenocytes isolated from CSC compared to SHC mice at 0.1 µM CORT. Interestingly, while relative delta cell viability was also significantly dependent on factor CORT in both SHCas and CSCas mice (Fig. 4D, Table 1), this parameter was significantly reduced at 0.1, 0.5 and 5 µM CORT compared to respective 0 µM CORT only in the SHCas, but not CSCas mice. Moreover, a significantly higher relative cell viability was found in CSCas vs. SHCas splenocytes at 0.05, 0.1, 0.5 and 5 µM CORT conditions, indicating the development of GC insensitivity exclusively in splenocytes from CSCas mice.

## Discussion

In the present study, we confirm previous findings^1^ by showing that exposure to 19 days of CSC does not affect spleen size, splenocyte (re)activity and spleen cell sensitivity to CORT, when significant wounding during stressor exposure is absent. We further extend these findings by showing that abdominal surgery prior to CSC exposure, in line with what we recently reported for bite wounds during CSC exposure^1^, promotes splenomegaly, basal and LPS-induced *in vitro* splenocyte activation and insensitivity towards GC in *in vitro* LPS-treated splenocytes. GC insensitivity is indicated by the inability of isolated and *in vitro* LPS stimulated splenocytes to respond to the immunosuppressive effects of CORT with a decrease in cell viability. Interestingly, the inability of isolated splenocytes from operated CSCas mice to respond to the inhibitory effects of GC was only detectable in the presence, but not in the absence, of LPS, which holds also true for splenic GC resistance as a consequence of bite wounds during CSC^1^.

We and others have repeatedly shown in mice that chronic psychosocial stressors promote development of GC insensitivity of isolated and *in vitro* LPS-stimulated splenocytes, accompanied by splenomegaly and splenic immune activation^2,9,10,16,26^. However, just recently we identified bite wounds as critical factor in the development of social stress-induced splenomegaly and splenic GC insensitivity, as well as facilitating factor in terms of social stress-induced spleen cell activation^1^. In the present study we provide now first evidence that also wounding induced by abdominal surgery one week prior to stressor exposure is able to promote CSC-induced splenomegaly, spleen cell activation and splenic GC insensitivity. As the latter is accompanied by increased susceptibility to endotoxic shock in mice^18^, this data from a translational point of view suggests that planned (i.e. surgery) or unplanned (i.e. physical trauma, injury, emergency surgery) wounding in combination with chronic stress represents a potential risk factors for surgical complications related to an overshooting immune response.

Mice exposed to 19 days of CSC without prior surgery had an average bite score of 14 out of possible 300, indicating that the severity of bite wounds in this group was relatively low. For comparison, CSC mice that develop moderate to severe splenomegaly, basal and LPS-induced *in vitro* splenocyte activation and insensitivity towards GC in *in vitro* LPS-treated splenocytes scored between 20 and 100 on our novel bite score scale^1^. Consequently, CSC mice in the present study did not develop an increase in spleen weight. In line with these and previous^1^ findings, the *in vitro* cell viability of isolated splenocytes from CSC mice was comparable to respective SHC mice under both basal and LPS-stimulated conditions, as was their delta response to LPS (LPS – basal), all in the absence of CORT. Isolated and *in vitro* LPS-stimulated splenocytes of CSC mice that did not undergo surgery before did further not develop any signs of GC insensitivity, which again is what we would have predicted^1^. Strikingly, CSCas mice, which underwent abdominal surgery one week prior to the start of the stress protocol, developed a pronounced splenomegaly when compared to both SHCas and CSC mice, although the total number of splenocytes did not differ from any of the other groups. The latter is again in line with previous findings showing splenomegaly despite an unaffected overall spleen cell count in bitten CSC mice^1^. Previous studies using fluorescence-activated cell sorting (FACS) further revealed that the unaffected splenic cell counts were accompanied by reduced numbers of smaller CD19^+^ and CD3^+^, but increased numbers of larger CD11b^+^ cells^1,7,27^. Although we did not analyze splenic cell composition in the present study, previous own work^1^ and studies using the SDR model^13^ demonstrating that splenic GC insensitivity is mediated exclusively by CD11b^+^ cells, support the hypothesis that splenomegaly seen in the CSCas group might also be due to increased numbers of CD11b^+^ splenocytes. However, whether the latter are also main mediators in the development of splenomegaly and splenic *in vitro* GC insensitivity in CSCas mice needs to be addressed in future studies. Accordingly, only splenocytes isolated from GC insensitive CSCas mice did not respond with a decreased delta cell viability (LPS minus basal, 0 CORT set to 100%) when *in vitro* LPS-stimulated and treated with increasing CORT concentrations. Moreover, the fact that in the absence of CORT an exaggerated cell viability under both basal and LPS-stimulated conditions, as well as an increased delta cell viability (LPS minus basal) was only detected in CSCas mice, further argues for an increased presence of CD11b^+^ splenocytes in this experimental group. Although it was demonstrated in a previous study that a mild stress-induced increase in (re)activity was also found in CD11b^+^ cell-depleted splenocytes isolated from CSC mice, main effects on this parameter were mediated by the CD11b^+^ fraction^1^. Further evidence for the conclusion that spleen changes seen in CSCas mice with prior abdominal surgery and non-operated CSC mice receiving bite wounds during stressor exposure^1^ share the same underlying mechanisms is provided by the fact that in both experimental groups splenic *in vitro* GC insensitivity can only be detected in the presence of LPS. The fact that this also holds true for *in vitro* splenic GC insensitivity following exposure to the SDR paradigm^10^ even argues for a general and stress paradigm independent phenomenon that occurs when chronic stress is combined with any kind of planned or unplanned wounding.

Taken together, the results of the present study demonstrate that physical injury induced by abdominal surgery prior to chronic stressor exposure in male mice is able to promote stress-induced splenomegaly, spleen cell (re)activation and splenic GC insensitivity. Together with previous findings, our results further suggest that physical injury in general is critically required for chronic stress-induced spleen effects, but that the timing of physical injury in the context of chronic stress only plays a minor role. However, the underlying mechanisms still need to be elucidated. From a translational perspective, these findings set the stage for a number of clinical studies investigating in a first step whether surgical complications are cumulating in patients with a history of life stress or with increased fear of the diagnosis, planned intervention or its outcomes.

## References

[1] Foertsch S (2017). Splenic glucocorticoid resistance following psychosocial stress requires physical injury. Scientific reports.

[2] Reber SO (2007). Adrenal insufficiency and colonic inflammation after a novel chronic psycho-social stress paradigm in mice: implications and mechanisms. Endocrinology.

[3] Langgartner, D., Fuchsl, A. M., Uschold-Schmidt, N., Slattery, D. A. & Reber, S. O. Chronic subordinate colony housing paradigm: a mouse model to characterize the consequences of insufficient glucocorticoid signaling. *Frontiers in psychiatry* **6**, 10.3389/fpsyt.2015.00018 (2015). 10.3389/fpsyt.2015.00018 PMC4337237 25755645

[4] Reber SO (2016). Chronic subordinate colony housing paradigm: A mouse model for mechanisms of PTSD vulnerability, targeted prevention, and treatment-2016 Curt Richter Award Paper. Psychoneuroendocrinology.

[5] Haffner-Luntzer, M. *et al*. Chronic psychosocial stress compromises the immune response and endochondral ossification during bone fracture healing via β-AR signaling. Proceedings of the National Academy of Sciences, 201819218, 10.1073/pnas.1819218116 (2019). 10.1073/pnas.1819218116 PMC6486758 30948630

[6] Langgartner D, Palmer A, Rittlinger A, Reber SO, Huber-Lang M (2018). Effects of Prior Psychosocial Trauma on Subsequent Immune Response After Experimental Thorax Trauma. Shock (Augusta, Ga.).

[7] Schmidt D, Peterlik D, Reber SO, Lechner A, Mannel DN (2016). Induction of Suppressor Cells and Increased Tumor Growth following Chronic Psychosocial Stress in Male Mice. PloS one.

[8] Friedrich-Carl, W. *Kleines Vademecum haematologicum Nordmark: eine Einführung in die Blutzellkunde*. (Nordmark Arzeimittel GmbH (1984).

[9] Sheridan JF, Stark JL, Avitsur R, Padgett DA (2000). Social disruption, immunity, and susceptibility to viral infection. Role of glucocorticoid insensitivity and NGF. Annals of the New York Academy of Sciences.

[10] Avitsur R (2003). Expression of glucocorticoid resistance following social stress requires a second signal. J Leukoc Biol.

[11] Avitsur R, Stark JL, Dhabhar FS, Padgett DA, Sheridan JF (2002). Social disruption-induced glucocorticoid resistance: kinetics and site specificity. Journal of neuroimmunology.

[12] Avitsur R, Stark JL, Sheridan JF (2001). Social stress induces glucocorticoid resistance in subordinate animals. Hormones and behavior.

[13] Stark JL (2001). Social stress induces glucocorticoid resistance in macrophages. American journal of physiology. Regulatory, integrative and comparative physiology.

[14] Engler H, Bailey MT, Engler A, Sheridan JF (2004). Effects of repeated social stress on leukocyte distribution in bone marrow, peripheral blood and spleen. Journal of neuroimmunology.

[15] Hanke ML, Powell ND, Stiner LM, Bailey MT, Sheridan JF (2012). Beta adrenergic blockade decreases the immunomodulatory effects of social disruption stress. Brain, behavior, and immunity.

[16] Sandra Foertsch, Stefan O. Reber. The role of physical trauma in social stress-induced immune activation. *Neuroscience & Biobehavioral Reviews* **113**, 169–178 (2020). 10.1016/j.neubiorev.2020.02.025 32109454

[17] Engler H, Engler A, Bailey MT, Sheridan JF (2005). Tissue-specific alterations in the glucocorticoid sensitivity of immune cells following repeated social defeat in mice. Journal of neuroimmunology.

[18] Quan N (2001). Social stress increases the susceptibility to endotoxic shock. Journal of neuroimmunology.

[19] Bailey MT, Engler H, Powell ND, Padgett DA, Sheridan JF (2007). Repeated social defeat increases the bactericidal activity of splenic macrophages through a Toll-like receptor-dependent pathway. American journal of physiology. Regulatory, integrative and comparative physiology.

[20] Avitsur R, Kavelaars A, Heijnen C, Sheridan JF (2005). Social stress and the regulation of tumor necrosis factor-α secretion. Brain, Behavior, and Immunity.

[21] Stark JL, Avitsur R, Hunzeker J, Padgett DA, Sheridan JF (2002). Interleukin-6 and the development of social disruption-induced glucocorticoid resistance. Journal of neuroimmunology.

[22] Foertsch, S. *et al*. Sensory contact to the stressor prevents recovery from structural and functional heart damage following psychosocial trauma. *Brain, Behavior, and Immunity*, 10.1016/j.bbi.2019.05.013 (2019). 10.1016/j.bbi.2019.05.013 31085218

[23] Langgartner, D.*et al*. Individual differences in stress vulnerability: The role of gut pathobionts in stress-induced colitis. *Brain, behavior, and immunity*, 10.1016/j.bbi.2016.12.019 (2016). 10.1016/j.bbi.2016.12.019 28012830

[24] Reber SO, Obermeier F, Straub RH, Veenema AH, Neumann ID (2008). Aggravation of DSS-induced colitis after chronic subordinate colony (CSC) housing is partially mediated by adrenal mechanisms. Stress (Amsterdam, Netherlands).

[25] Singewald GM, Nguyen NK, Neumann ID, Singewald N, Reber SO (2009). Effect of chronic psychosocial stress-induced by subordinate colony (CSC) housing on brain neuronal activity patterns in mice. Stress (Amsterdam, Netherlands).

[26] Füchsl AM, Neumann ID, Reber SO (2014). Stress Resilience: A Low-Anxiety Genotype Protects Male Mice From the Consequences of Chronic Psychosocial Stress. Endocrinology.

[27] Wendt, F.-C. *Kleines Vademecum haematologicum Nordmark: eine Einführung in die Blutzellkunde*. (Stormarn-Verlag (1986).

